# Endovascular repair of giant primary iliac venous aneurysm using inverted iliac artery stent grafts

**DOI:** 10.1016/j.jvscit.2025.101941

**Published:** 2025-08-06

**Authors:** Yonglong Wang, Mingxian Zhao, Zhengfei Li, Ying Liang, Yongxia Wu, Xiaojun Shu

**Affiliations:** a The First School of Clinical Medicine, Lanzhou University, Lanzhou, Gansu, China; b Department of Vascular Surgery, Qinghai Province Cardiovascular and Cerebrovascular Disease Specialist Hospital, Xining, Qinghai, China; c Department of Vascular Surgery, General Surgery, The First Hospital of Lanzhou University, Lanzhou, Gansu, China; d Psychosomatic Medicine, Shanghai General Hospital Jiuquan Hospital (The People's Hospital of Jiuquan), Jiuquan, Gansu, China; e The Second Hospital & Clinical Medical School, Lanzhou University, Lanzhou, Gansu, China

**Keywords:** Iliac vein aneurysm, Endovascular repair, Stent graft

## Abstract

Primary iliac venous aneurysms are rare, with etiologies linked to congenital and hemodynamic factors. We report a 60-year-old woman with a 75-mm iliac venous aneurysms treated with inverted iliac artery stent grafts. At the 1-year follow-up, stent patency and symptom resolution were confirmed, although mural thrombosis mandated prolonged anticoagulation. Endovascular repair offers minimal invasiveness but requires long-term validation.

Iliac venous aneurysms (IVAs) are exceptionally rare, with the iliac system representing the least common location for venous aneurysms.^1^ Primary IVAs, defined by the absence of arteriovenous fistulae or congenital anomalies, are particularly uncommon and may arise from congenital wall weakness or hemodynamic stressors such as May-Thurner syndrome.^1^, ^2^, ^3^ Although often asymptomatic, IVAs pose significant risks of pulmonary embolism, rupture, or mass effect symptoms.^4^^,^^5^

Owing to their rarity, management lacks consensus. Endovascular repair offers a minimally invasive alternative, yet concerns persist regarding long-term patency, sizing challenges in compliant venous systems, and mandatory anticoagulation.^6^, ^7^, ^8^ We report a case of endovascular exclusion of a giant (75-mm) primary external iliac vein aneurysm using inverted iliac artery stent grafts. This approach highlights an innovative strategy for managing complex IVAs while underscoring unresolved challenges in antithrombotic management. The patient provided consent for this case report.

## Case report

**Fig 1:**
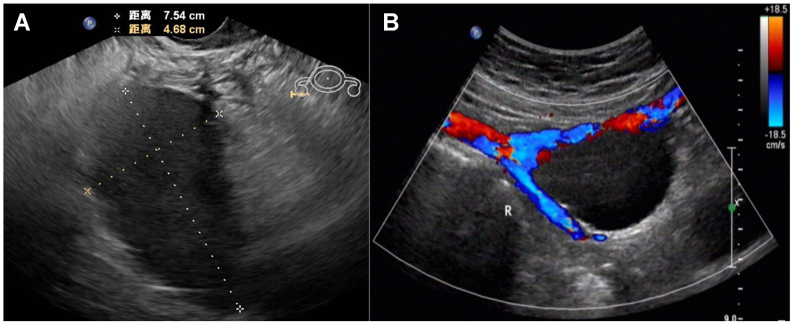
**(A)** Color Doppler ultrasound examination demonstrates a 75.4 mm × 46.8 mm anechoic mass adjacent to the right iliac vessels. **(B)** Corresponding Doppler image confirms the absence of internal flow signals within the lesion.

**Fig 2:**
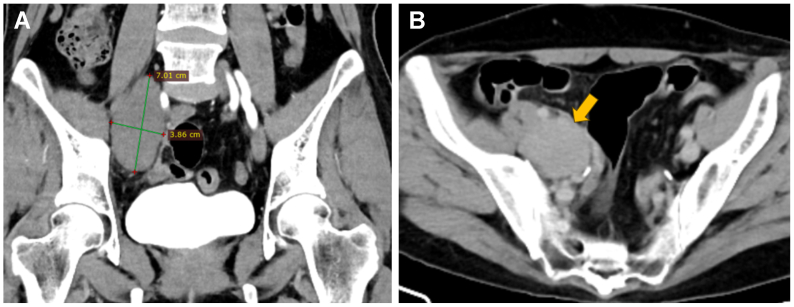
Contrast-enhanced computed tomography scan. **(A)** Coronal reconstruction reveals a large space-occupying lesion contiguous with the right external iliac vein. **(B)** Axial view during the venous phase demonstrates mild enhancement of the aneurysm (*arrow*).

A 60-year-old woman presented with a 1-year history of intermittent right lower abdominal pain. Physical examination revealed no significant findings. The patient had no prior history of pelvic surgery or trauma, and her coagulation function was normal. Color Doppler ultrasound examination revealed a 75.4 mm × 46.8 mm anechoic area adjacent to the right iliac vessels, with no flow signal (Fig 1). A contrast-enhanced computed tomography scan confirmed the diagnosis of a right external iliac vein aneurysm (Fig 2). When the patient declined open surgical repair, she was managed with endovascular therapy.

**Fig 3:**
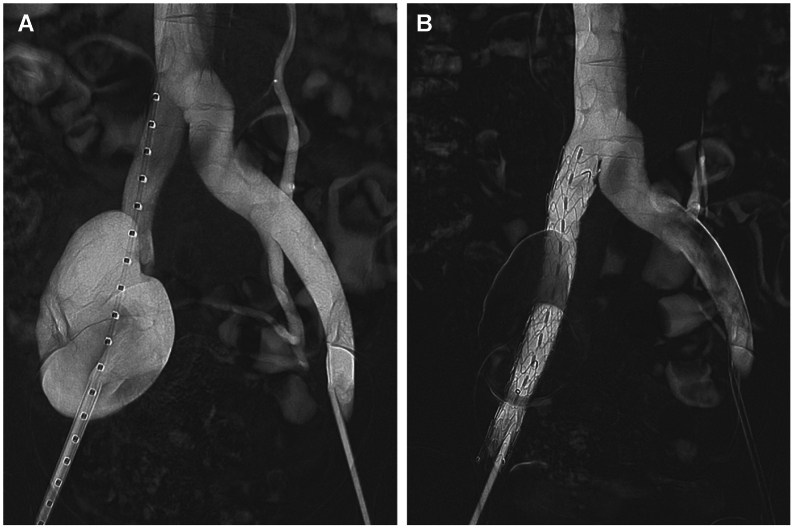
**(A)** Antegrade venography via the right common femoral vein sheath shows saccular aneurysmal dilation of the proximal right external iliac vein. **(B)** Completion venography after deployment of two inverted iliac artery stent grafts confirms successful exclusion of the aneurysm with no contrast extravasation and preserved flow through the stented segment.

Under local anesthesia, bilateral 6F sheaths were placed percutaneously in the common femoral veins. Antegrade venography performed via the sheaths confirmed the diagnosis of a saccular aneurysm in the proximal right external iliac vein (Fig 3, *A*). To achieve optimal exclusion, two iliac artery extension stents (IE14-24-080/IE14-16-080; Lifetech Scientific Co., Ltd., Shenzhen, China) were prepared using an intentional inversion technique. (1) Each stent graft was fully deployed on a sterile field under controlled conditions. (2) The device was then manually inverted 180° along its longitudinal axis, reorienting the tapered configuration such that the wide diameter end was positioned toward the planned proximal landing zone. (3) The inverted stent graft was reloaded carefully into its original delivery sheath (16F), ensuring minimal material stress. Under fluoroscopic guidance, the inverted stent grafts were sequentially deployed within the right external iliac vein. The intentionally repositioned wide diameter end was anchored proximally at the iliocaval junction, while the narrow diameter end extended distally, conforming to the natural venous taper. Completion venography confirmed aneurysm exclusion without endoleak or collateral filling (Fig 3, *B*).

**Fig 4:**
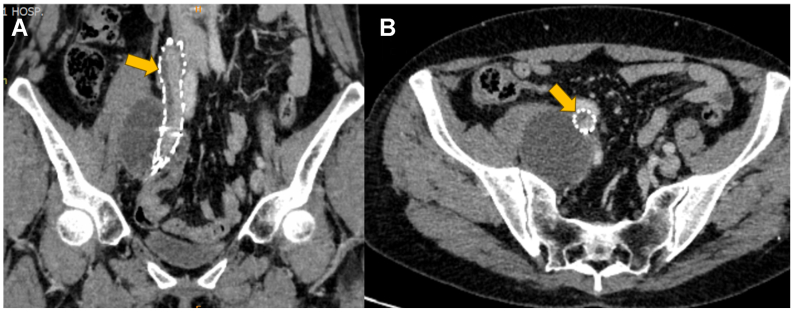
**(A)** Coronal view shows contrast opacification within the patent stent graft lumen. *Arrow* indicates mural thrombus formation along the stent wall. **(B)** Axial view confirms the presence of mural thrombus (*arrow*) within the stent lumen.

Postoperatively, anticoagulation with low-molecular-weight heparin and pneumatic compression therapy were initiated. The patient had an uncomplicated recovery and was discharged on postoperative day 3. She was prescribed continued oral rivaroxaban and instructed to wear compression stockings. At the 1-year follow-up, the patient remained asymptomatic, reporting no abdominal pain or lower extremity swelling. A surveillance contrast-enhanced computed tomography scan confirmed that the stent grafts remained patent and well-positioned within the right external iliac vein (Fig 4). However, it revealed mural thrombus lining the stent lumen, despite therapeutic rivaroxaban levels. Given the significant risk of thrombus propagation, occlusion, or embolization, the decision was made to continue indefinite therapeutic anticoagulation with rivaroxaban. The patient provided written informed consent for publication. This study was approved by the Ethics Committee of The First Hospital of Lanzhou University [LDYYLL2024-481].

## Discussion

The management of IVAs lacks consensus owing to their rarity. Intervention is indicated for symptomatic/complicated IVAs or diameter exceeding three times that of the adjacent normal venous segment.^6^^,^^9^ Endovascular repair offers less invasiveness, minimal blood loss, and faster recovery.^4^^,^^9^ Optimal oversizing of iliac venous stents is recommended at 20% to 25% relative to the landing zone diameter to mitigate migration risk.^9^ The strategic selection of an inverted iliac artery extension stent graft in this iliac venous aneurysm conferred distinct advantages. (1) Its covered design enabled complete endoluminal exclusion of the aneurysmal sac. (2) The tapered configuration, when intentionally inverted, accommodated the physiological luminal reduction from the proximal common iliac vein to the distal external iliac vein.

Optimal antithrombotic therapy after venous stenting lacks robust evidence.^7^ Reported thrombosis rates after venous covered stenting range from 5.7% to 11.5% acutely and 7.7% to 8.6% during follow-up.^10^^,^^11^ For IVA repair, thrombosis risk is heightened by stent caliber, low venous flow, and residual sac stasis. Our patient developed mural thrombus despite therapeutic rivaroxaban, underscoring this risk. The International Delphi Consensus recommends 3 or more months anticoagulation for nonthrombotic iliac vein stenting, with extended therapy based on risk factors.^7^ Given our finding of mural thrombus, indefinite rivaroxaban is prudent to prevent occlusion or embolism, aligning with other reports.^4^^,^^8^

## Conclusions

Endovascular repair using inverted iliac artery stent grafts is feasible for giant primary IVAs, demonstrating good short-term results. It provides a minimally invasive alternative to open surgery, advantageous for high-risk patients. However, higher endovascular reintervention rates and our case's mural thrombus (requiring indefinite anticoagulation) highlight key limitations. The inverted technique aims to optimize fixation and sizing but requires further study. Careful patient selection, precise oversizing, mandatory long-term anticoagulation, and rigorous surveillance are crucial. Multicenter studies with extended follow-up are needed to define endovascular roles, refine techniques (including stent inversion), and optimize antithrombotic regimens for these rare lesions.

## Funding

Funded by the 10.13039/501100018554Gansu Provincial Science and Technology Program. No.21JR7RA358.

## Disclosures

None.
